# Resilience-focused debriefing: addressing complexity in interprofessional simulation-based education—a design-based research study

**DOI:** 10.1186/s41077-025-00352-4

**Published:** 2025-04-29

**Authors:** Torben Nordahl Amorøe, Hans Rystedt, Lena Oxelmark, Peter Dieckmann, Paulin Andréll

**Affiliations:** 1 Department of Anesthesiology and Intensive Care Medicine, Institute of Clinical Sciences at the Sahlgrenska Academy, University of Gothenburg, Gothenburg, Sweden; 2 Department of Research, Education & Innovation, Simulation Centre West, Region Västra Götaland, Sahlgrenska University Hospital, Diagnosvägen 10, SE- 416 85, Gothenburg, Sweden; 3 Institute of Health and Care Sciences, the Sahlgrenska Academy, University of Gothenburg, Gothenburg, Sweden; 4 Copenhagen Academy for Medical Education and Simulation (CAMES), Center for Human Resources, Capital Region of Denmark, Herlev Hospital, Herlev, Denmark; 5 Department of Quality and Health Technology, University of Stavanger, Stavanger, Norway; 6 Institute of Public Health, University of Copenhagen, Copenhagen, Denmark; 7 Department of Anaesthesiology and Intensive Care Medicine/Pain Center, Region Västra Götaland, Sahlgrenska University Hospital Östra, Gothenburg, Sweden

**Keywords:** Interprofessional education, Safety-II, Resilient health care, Patient simulation, Appreciative inquiry, Solution-focused approach

## Abstract

**Background:**

Healthcare students are taught teamwork and collaboration through interprofessional simulation-based education (IPSE). However, the complex nature of healthcare and the ability to react resiliently to the unexpected is usually not actively addressed. This study explores how complexity and resilience can be addressed in IPSE debriefing for pre-graduate healthcare students.

**Methods:**

A focus group of nine facilitators in an IPSE course for nursing and medical students was introduced to the characteristics of complex systems, Safety-II, solution-focused approach, and appreciative inquiry. In five iterations, the facilitators discussed how these theories and methods could be applied, tested, evaluated, and adjusted in debriefings supported by video clips of their own debriefings. Video recordings of debriefings (*n* = 56) and focus group interviews (*n* = 6) were collected. Focus group interviews were transcribed and reviewed to explore the basis for final recommendations.

**Results:**

Facilitators identified and tested 22 debriefing techniques that potentially could address complexity and resilience in IPSE. In total, 17 of the tested techniques were found to be able to make students aware of the complex nature of interprofessional teamwork and collaboration in acute dynamic healthcare situations, their existing capacities for resilience, potentially increasing their capacity for resilience.

**Conclusions:**

Learning needs around resilience and complexity could be addressed successfully in IPSE debriefings, but further studies are needed to assess the effect of resilience-focused debriefing techniques on teamwork in IPSE.

## Background

Quality of care and patient safety relies on the ability of interprofessional healthcare teams to collaborate effectively under complex and constantly changing conditions. Interprofessional simulation-based education (IPSE) has been increasingly used as a method for preparing healthcare students and professionals for efficient teamwork and collaboration in order to increase patient safety [1, 2].

This study is premised on theories in that healthcare is a complex adaptive system composed of multiple elements including patients, healthcare staff, organizations, policies, and technology [3]. These elements act interdependently, adapt, and self-organize in a dynamic system exhibiting emergent behaviours, making it difficult to predict outcomes [3–5]. Interprofessional healthcare teams can be considered smaller complex systems [6]. This perspective on complexity implies the need for resilience, i.e. “the capacity to adapt to challenges and changes at different system levels, to maintain high quality care” [7]. For example, this may be required when a department needs to manage staff absences due to illness or when a patient’s condition suddenly deteriorates. Hollnagel and colleagues claim that patient safety can be increased if healthcare develops its capacity for resilience [8]. According to the authors, to achieve this, it is necessary not only to analyse what goes wrong and try to avoid mistakes (labelled Safety I) but also to analyse what goes right and learn from successes (Safety-II) [9]. Further, it has been suggested that simulation-based education may be employed to improve the awareness of complexity and develop resilient capacities [5, 10, 11].

IPSE training involves interprofessional teams managing medical challenges in full-scale simulation scenarios followed by post-simulation debriefings. The debriefing is seen as essential for participants to learn from the scenarios [12]. Debriefings are often scripted, commonly incorporating variations of Steinwachs’ 3 phases of description, analysis, and application [13]. Debriefings allow participants to make sense of their experience and learn from it. Generally, it is recommended to ask participants to identify what went well [12], but primary focus is often put on identifying knowledge and performance gaps, for example making corrections if guidelines/algorithms are not followed [11, 14].

While much simulation training closely follows pre-established guidelines and algorithms, it has been criticised for not accounting for the fact that simulation training must also consider the complex nature of healthcare systems and the ability of teams to respond resiliently, i.e. to adapt to and recover from unexpected and unpredictable events [5, 11]. Thus, current techniques often prioritise error correction and adherence to guidelines, whereas resilience-focused approaches emphasise adaptive behaviours and learning from success. For example, the following illustrate a corrective approach: “You did not adhere to the guideline during your treatment, which I think is problematic. What could have helped you to stick closer to the guideline?” In contrast, the following illustrate a focus on adaptation and recovery: “You deviated from the guideline and also recovered from that. What helped you to notice the deviation and figure out how to get back on track?” Previous findings [15] indicate that focusing solely on pre-defined guidelines and algorithms is insufficient; rather, unpacking complexity and highlighting how solutions are achieved and challenges overcome is also important.

To learn from success, methods such as solution-focused approach and appreciative inquiry [16, 17] have been put forward [9], specifically in post-simulation debriefings [12, 18]. These methods deal with exploring strengths, how challenges are overcome and the positive setting of concrete, achievable goals in order to develop motivation for lasting change in organizations, teams, and individuals alike. In addition, Johansson et al. showed how video could serve as a promising tool to promote more in-depth reflections during debriefings, since it enables a re-actualization of scenarios on a detailed level that preserves the complexity as a shared point of departure for reflection-on-action.

There is a lack of empirical studies on how post-simulation debriefings in IPSE can be designed to address the complexity and resilience involved in healthcare work and how this can be achieved through learning from success. Against this background, the present study explores how debriefing can highlight complexity and how resilience can be addressed at the team level. It also suggests debriefing techniques to achieve this in interprofessional simulation-based education for pre-graduate healthcare students.

## Methods

### Research design

The aim of this study was to develop, test, and evaluate theoretically and empirically founded debriefing techniques for IPSE that address the complexity of healthcare work and the need for resilient capacities.

This study was designed according to principles for design-based research (DBR) [19]. DBR emphasises research in real-life settings to understand and iteratively develop educational practice, producing results that are useful for educators and learners alike. As part of this project in a previous study [15], we explored how theories of complexity and resilience affect IPSE from the perspective of facilitators.

### Research setting

**Fig. 1:**
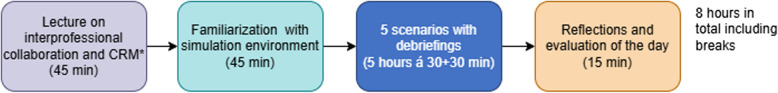
Program for the interprofessional simulation-based education course. *Crisis resource management

In their final semester, pre-graduate nursing and medical students from the University of Gothenburg in Sweden participated in a mandatory 1-day simulation course at the Simulation Centre in Sahlgrenska University Hospital (Fig. 1) [15]. The objective of the course was to enhance students’ communication and collaboration skills, enabling them to care for acutely ill patients effectively and safely.

Each scenario was designed to incorporate common acute medical-technical challenges (chronic obstructive pulmonary disease, hypoglycaemia, postoperative bleeding, postoperative sepsis, and ketoacidosis); nursing and crisis resource management (CRM) aspects; and ABCDE (airway, breathing, circulation, disability, exposure/environment), e.g. “speaking up” [20] covering specific challenges to both professions. Scenarios varied in severity, onset, presence of family, and possibility of assistance. In debriefings, a 3-phase model of description-analysis-application was followed.

Roughly 200 students participated in the course each term, organized into groups of around eight (four nursing students, four medical students). While 3–5 students actively participated in each scenario, the remaining students observed a live video feed from another room. Debriefing was facilitated by one nurse and one physician.

### Participants and sampling

Out of a total of 20 invited facilitators that conducted debriefings in the IPSE course nine volunteered to participate in a single focus group that were interviewed on numerous occasions. The average age was 44 years (range 32–61), seven women and two men. Six were specialist doctors, and three were specialist nurses. On average, the facilitators had been facilitating for 4.5 years (range 2–8). Throughout the research project, these facilitators facilitated an average of 22 scenarios on the IPSE course (range 8–35). All facilitators had previously participated in simulation instructor courses lasting at least 3 days.

### Intervention

The facilitators were introduced to the characteristics of complex systems (unpredictability, emergence, actors’ adaptation and self-organizing interdependently, non-linear causality) and the concept of organizational resilience including the idea of bouncing back from setbacks. Further, they were introduced to techniques from Safety-II, solution-focused approach and appreciative inquiry (constructionist principle, exploring what went well, exceptions to past failures, contrasting problem and solution-focused approaches, magic question, setting positive goals that are small, concrete, and achievable).

The facilitators were asked to discuss how these theories and methods could be applied to IPSE debriefings particularly and to agree on changes to their current debriefing practice that they would focus on trying out and evaluating in upcoming debriefings.

**Fig. 2:**
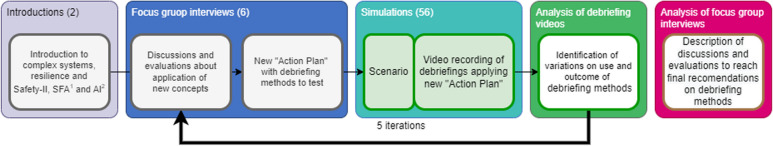
Data collection and analysis process. The blue and green boxes represent one iteration. The red box represents analysis of focus group interviews. ^1^Solution-focused approach. ^2^Appreciative inquiry

All debriefings (56) were video recorded. After each IPSE course day, each debriefing recording was reviewed by the first author. Dialogue was transcribed if it concerned a specific technique and alluded to complexity and/or resilience. Video clips were chosen if they could show variations of the use of techniques and resulting dialogue including clips seemingly contradicting the expected effect as discussed in the previous focus group interview. In subsequent focus group interviews, the facilitators discussed and evaluated the execution of the techniques in the “action plan” in order to keep, adjust, or dismiss the techniques. This process was iterated five times (Fig. 2).

### Data collection

Data was collected from August 2017 to June 2018. Data collection took place in an iterative process (Fig. 2). The primary data consisted of the video recordings of the six focus group interviews (approximately 11 h).

The interviews were directed by the first author, who was a PhD-student, specialist physician in anaesthesiology and intensive care with 11 years of facilitator experience. The video format was chosen to identify which debriefing video clip was being discussed. One of the co-authors (alternating) took observational notes to assist the analysis. The notes focussed on discussions about complexity and the tested techniques. The first two focus group interviews were initiated after a presentation of the intervention. Semi-structured interview guides comprised questions on how the facilitators applied, tested, and evaluated the suggested techniques (Supplementary Material 1 in [15]).

### Data analysis

After all the interviews were completed, the focus group interviews were transcribed verbatim by the first author. The analysis took place in four stages:

Firstly, all discussions on specific *debriefing techniques* set out to be tested in the action plans (e.g. “What did you do to overcome or resolve the situation?”) were identified and labelled accordingly (e.g. “Success” and “Challenges”) using Microsoft Excel. Some debriefing techniques discussed by the focus group were deemed to not clearly be related to complexity and resilience and were therefore excluded.

Secondly, for each identified technique, discussions on the outcomes of testing (*evaluations*) were identified (e.g. using “overcome” seemed to generate rich discussions, whereas using “strategies” required further testing and discussion in the next iteration). Emphasis was placed on presenting techniques where facilitators reached both positive and negative evaluations and where disagreement was present. A condensed summary of discussions for each debriefing techniques along, with its evaluation, was produced.

Thirdly, the tested debriefing techniques and their corresponding evaluations were grouped according to what types of *problems* that the techniques were considered to be solutions to regarding addressing complexity and resilience. This grouping was inspired by previous research findings [15]. In the fourth stage, the findings were synthesised into a narrative illustrating the discussions about benefits and drawbacks using the debriefing techniques to address the problems. All authors participated in each stage of the analysis.

## Results

The facilitators identified, tested, and adjusted 22 *debriefing techniques* presented as potential solutions to *problems* addressing complexity and resilience in IPSE and their corresponding *evaluations* (Table 1). The problems are organised in three overarching problems with five more specific problems. Further, the results include the facilitators’ motifs for each potential solution including their reasons for abandoning some (numbers in text refer to the techniques shown in Table 1). Seventeen of the techniques were adopted. Two of these techniques were recommended if time and workload constraints otherwise permitted. Five techniques were rejected. The facilitators were given the opportunity to arrange the debriefing as they liked, but the three-phase model of description-analysis-application was kept.

**Table 1:** Problems addressing complexity and resilience in IPSE, the suggested potential solutions as debriefing techniques and their corresponding evaluations

**Problems**		**Debriefing techniques**	** + / − **	**Evaluations**
**I Being unaware of complexity**				
1 How should debriefing topics chosen when addressing complexity?	1.1	Using plus/delta	+ / −	Considered valuable, but too time consuming
	1.2	Only focusing on the team-level	−	Individual focus impossible to leave out
2 How do the students get a clearer picture of the complexity within the scenario?	2.1	Exploring why things turned out the way they did	+	Shows students’ perspective shows potentially multiple contributing factors
	2.2	Inquiring into the consequences for the team or the patient	+	Avoids only looking at adherence to guidelines, but helps students see what the actual outcomes of decisions and actions were
	2.3	Exploring expressions of “messiness”	+	An expression of complexity helps students clarify what is behind this and normalise
	2.4	Exploring basis for “uncertainty”	+	Potentially an expression of complexity helps students clarify what is behind this and normalise
	2.5	Asking for “adaptations”	−	As an open question this does not seem to make sense for students
	2.6	Exploring different perspectives by asking chain of follow-up questions	+	Follow questions to a particular student by asking the team for perspectives, returning to the student to get reflections on team’s perspectives. Secures interprofessional perspectives and reinforce learning
**II Not recognizing resilience**				
3 How can the students learn from success?	3.1	Asking for what students did well or were good at as an open question	−	Students uncomfortable focused on standards that they did not achieve and therefore cannot produce anything they were good at
	3.2	Asking for “contributions”	+	Easier for students to answer, less pressure in a contribution, brings forth multiple contributions more easily not just one right one
	3.3	Exploring how success was achieved	+	Not necessarily just because standards were followed, helps students become aware of strengths
	3.4	Using video	+ / −	Considered very beneficial, but too time consuming and strenuous for facilitator
4 How can the students learn from struggles, perceived failures or bouncing back	4.1	Informing about messiness and resilience in introduction of the day	+	Important to prepare students for idea that they may experience uncertainty and “messiness”, and that this is normal
	4.2	Asking what the students would have done differently as an open question	−	Students compare to standard going straight to the one right thing that should have been done, without exploring multiple perspectives and variations
	4.3	Asking for "challenges" as an open question	+	Students more readily reflect on what actually happened within the scenario
	4.4	Asking for how challenges were overcome	+	This is KEY. Successful or attempted. This potentially highlights already existing capacity for resilience
	4.5	Exploring several possible solutions	+	Acknowledges that there may not be only one way of doing things especially if conditions vary
	4.6	Avoiding learning from hindsight	+	Avoids accepting “insights” from information the students only gained later in the scenario
	4.7	Obtaining positive goals	+	Above all helpful when students did not succeed in an attempt or intention and after complexity and resilience has been explored. Then, ask how they would like to manage a similar situation in the future
	4.8	Asking what a “perfect” solution would look like	-	Aggravates students’ tendency to focus on what they perceived as failures
	4.9	Shortening analysis of positive aspects		If students are eager to analyse “negative aspects” it is acceptable to move on to these early on, since exploring perceived failures by exploring complexity and potentially reframing weaknesses is less threatening
**III Learnings potentially unclear**				
5 How can students achieve valuable learnings?	5.1	Probing for concrete strategies	+	Not accepting abstract answers by asking how learnings are planned to be carried out concretely provides clearer learnings and new objectives for the students

### Being unaware of complexity

An overarching problem addressed by the facilitators was that the students often were unaware of the complexity of situations. How then, should debriefing topics be chosen to address complexity? The plus/delta technique was suggested [12] to effectively identify important issues when exploring complexity (1.1). While they initially found it compelling, the facilitators rejected this technique because they found it too time-consuming and difficult to keep the students from starting to engage in negative aspects too early in the analysis phase. Instead, the facilitators reverted to balancing specified learning goals, facilitators’ observations, and students’ reactions when choosing issues to examine. As the facilitators adopted an organizational resilience perspective, the focus was initially on the team-level (1.2). However, this was rejected, as the facilitators argued that the individual students’ understanding of complexity and resilience was important for learning.

The facilitators observed that often self-criticism referred to failure to adhere to guidelines. As part of exploring the complexity of the situation, it became important to go beyond the simple explanation that guidelines were not followed. Thus, it was critical to identify whether several contributing factors led to why things turned out the way they did (2.1). On the one hand, this could consist of uncovering different perceptions, intentions, and strategies of the team members. On the other, it could relate to unexpected emergent events within the scenario, e.g. the team leader was on the phone when a team member needed to inform about blood pressure.

The facilitators recounted that analysis in what they considered “traditional” debriefings often stopped when it was concluded that a guideline was not adhered to. This was often followed by asking what the student should or would do instead. The facilitators found it important to keep analysing and see what the consequences were of adhering or not adhering to guidelines were within the scenario (2.2). According to the facilitators, the students should not *only* become able to evaluate their application of algorithms. “We want them to learn to take responsibility for solving situations, even if things do not go as expected” such as unintentionally not following guidelines strictly.

The facilitators found that the students often expected a sense of clarity from the very start all through the scenarios, evoking unrealistically high expectations among the students. They stated that students’ expressions of messiness and uncertainty should be seen as characteristics of a complex situation (2.3–4). Such expressions did not necessarily reflect lack of knowledge but should be seen as cues to explore complexity further. The facilitators emphasised that the students needed to become aware that the goal was not to free themselves of uncomfortable feelings stemming from not being in control but to work to gain control despite such feelings. The facilitators agreed that the students should be prepared to experience messiness and uncertainty by informing them about complexity in a pre-briefing (4.1).

An approach was adopted whereby a chain of follow-up questions was asked to bring forth different perspectives and thus explore complexity (2.6). First the facilitator would ask a student about a certain issue. Second, the rest of the team would be invited to give their perspectives. Third, the original student would be asked for a new reflection on what the team just said. The addition of returning to the first student was a new practice. This approach was intended to involve all students even if one person was at the centre, thus bringing forth interprofessional issues more clearly.

### Not recognizing resilience

Inspired by the Safety-II recommendation to learn from success, debriefing techniques were identified and tested to strengthen resilience capacities by exploring what went well. However, the usual practice of the facilitators asking *what did you do well* was abandoned (3.1). This question seemed to narrow the spotlight onto to what degree guidelines were followed. Instead, the facilitators adopted the practice of asking for contributions (3.2) directed to an individual or the team. The students more readily answered this question, and answers were more concrete and focused on how specific decisions and actions led to successful treatment. “It is evident that everyone has contributed with something” a facilitator said. Also, the word *contribute* fitted well with the idea of complexity acknowledging an outcome could rely on multiple contributing factors. Asking *how did you succeed* seemed to have the same effect as using *contribute* (3.3). The use of video-clips (3.4) to emphasise positive aspects was found to be very useful, although it was regarded as being too time consuming and as complicating the facilitators’ workload.

While the facilitators acknowledged that performance gaps could warrant need for corrections, they also found that perceived performance gaps were often not due to an individual’s or a team’s lack of knowledge or abilities. Even if individuals and teams adequately adjusted their actions to the uncertainty and messiness of the situation, it might take a while to acquire a sense of being in control. Therefore, perceived performance gaps would have to be examined, and might lead to reframing of perceived weaknesses.

According to the facilitators, their usual practice of asking if there was *anything that the students should like to do differently* (4.2) as an open question, often led students to only relate their performance to prescribed guidelines. Instead, asking for *challenges* (4.3) was found to be useful when dealing with perceived negative aspects or improvements. This generated a more focused attention to the difficulties occurring within the scenario, providing a stepping stone to address the complexity of the situation.

Questions on how the students handled or overcame their challenges (4.4) were an important way of making the students aware of their resilient capacities as they often seemed blind to their own abilities. A facilitator expressed the following:

When we talked about “succeeded”, it was in the context that there was something, actually going bad for them at first, but then this successful “turning” came about.

This approach led to reducing the students’ self-critique according to the facilitators, and the question rendered richer reflections than the usual follow-up question *Is there anything you would (or should) have done differently?* when performance gaps were identified.

The facilitators meant that neither in everyday clinical life nor in scenarios there are universal solutions but rather several alternative routes to successful outcomes. Therefore, they argued that exploring several alternative solutions (4.5) and reflecting on benefits and drawbacks should be allowed. It was found important to avoid false insights from hindsight (4.6), which was perceived as a trap for both students and facilitators, i.e. when students stated that they should have done something earlier despite not having had sufficient information at that point in time.

Especially when challenges in handling complexity were not overcome, the facilitators found it was particularly important to get students to formulate positive goals (4.7) in order to potentially increase their resilient capacity, either by exploring intentions or by asking what they would do if they could do the scenario over*.* However, asking for what a *perfect* solution would look like (4.8) seemed to fixate the students’ attention on how they did NOT meet the standards.

If the students were eager to talk about perceived negative aspects, the facilitators found it useful to allow this even if it was in the “positive” section of the analysis phase (4.9). This was found to be acceptable since addressing complexity and resilience was thought to be less threatening when addressing negative aspects.

### Learnings potentially unclear

The facilitators observed that students often came up with abstract strategies such as “communication is important”. While concreteness was important in general, it seemed even more important, when debriefings revolved issues of complexity. Thus, getting the students to formulate concrete actionable strategies when formulating goals was emphasised, especially in the application phase by using concretizing questions (5.1) A facilitator exemplified this when a student said “I shall dare to make speak ups”. “Ok, and what would that sound like?”, i.e. a “micro-simulation” in the debriefing. This also acknowledged how guidelines like CRM could be the very tools that may help manage complexity.

## Discussion

This research aimed at developing theoretically and empirically founded debriefing techniques [12] for IPSE that address complexity and resilience by focusing on learning from success. Using principles of DBR, we found and evaluated multiple debriefing techniques in IPSE that can make students aware of the complex properties of interprofessional teamwork in acute situations, and their capacities for resilience and potentially increase such capacities and thereby potentially secure a more comprehensive understanding of interprofessional teamwork.

The adopted debriefing techniques represent a mindset or perspective [15] that a simple corrective approach to simulation-based education might have shortcomings [5, 21–23]. This perspective acknowledges the reality of “muddling through” [24], i.e. the necessity of making decisions and acting in spite of uncertainty and lack of information. The adopted debriefing techniques attempt to secure the exploration of complexity by going beyond identifying and correcting perceived performance gaps [3, 4, 11, 22].

Although other debriefing techniques acknowledge the need to address complexity [14, 22], few techniques have resilience as the primary focus [11, 25]. The solution to handling situations that are described as complex and dynamic is often standardisation of processes, and that simulation should train these standardised processes (Gaba et al., 2001). The techniques of advocacy-inquiry and circular questions represent ways of exploring participants’ perspectives and complexity [14, 22]. While advocacy-inquiry primarily explores perspectives to expose individual’s erroneous reasoning, circular questions’ exploration of perspectives is explicitly designed to investigate complexity. This is achieved by having a third person, e.g. an observer openly reflect on team members’ interactions [22].

The emphasis on examining how challenges and perceived weaknesses were overcome within the scenario is one of the most important findings in this study. Rather than just correcting a perceived flawed behaviour, it is more valuable and motivating to highlight how initial mistakes and challenges were solved within the scenario [11, 15].

Recommending that students look more closely at what went well and making them aware of their strengths are common elements of debriefing practice [12, 25, 26]. These are usually applied in order to reinforce correct performance and to create an open atmosphere in preparation for examining what needs to be improved [26]. In line with our findings, Diaz-Navarro stresses exploration of success to understand performance variability and adjustments in complex situations [27].

It is an important finding in our study that the facilitators experienced it was easier for students to reflect on what transpired within the scenarios when asked about *contributions* and *challenges* than when asked what they did well and what they should have been done differently. Although the students’ reflections regularly referred to justifiable actions to overcome the situation at hand, this wording seemed to encourage them to formulate their experiences more freely by placing less emphasis on the ideal standard. In this way, it demonstrates how the phrasing of questions can either facilitate or constrain the students’ reflections in decisive ways, which aligns with Johansson’s findings [28].

In the endeavour to develop tools for healthcare staff to learn how to translate resilience into practice, Haraldseid-Driftland et al. found a number of learning principles that are parallel to our debriefing techniques in IPSE, such as using a collaborative approach, creating awareness of adaptive capacities, and sharing examples of good practice [29]. Our perspective on resilient healthcare and safety-II is in line with Bentley et al. which further raise the importance of focusing on what went well and how things actually played out within the scenario [25].

While previous research on using videos has shown mixed results [30–32], the facilitators found showing video of successful behaviours to be very useful providing an opportunity to deepen reflections on complexity and resilience. However, as the use of video takes time and put an extra workload on the facilitator, this technique is only recommended if sufficient time is available.

The facilitators clearly stated that guidelines/algorithms very well could contribute to resilience. Both deviating from and following guidelines can be means of managing complexity [15]. Also, the CRM points were originally designed to handle complexity and dynamism [33].

Implications of these findings are that facilitators in IPSE have a set of debriefing techniques that they can apply immediately to address complexity and resilience. This may provide students with relevant and successful strategies to handle the complexity of teamwork and potentially improve patient safety in future clinical life.

### Strengths and limitations

This study answers the calls for theory-driven research on simulation-based educational [34] and empirical studies exploring practical applications of complexity theory and organisational resilience [3, 35]. This study adds to the knowledge about using DBR to research and develop new simulation-based educational principles using the strengths and agility of the iterative process of planning, testing, and evaluating [19].

It is worth noting that this study was conducted in the context of pre-graduate nursing and medical students in a simulated environment. Therefore, it cannot be determined to what extent these findings are applicable to professionally experienced staff in real-life settings, highlighting the need for further research.

Measures to ensure trustworthiness and dependability in the analysis were undertaken [36]. The authors PA, HR, LO, and TNA reviewed the interview guides (Supplementary Material 1 in [15]). Multiple meetings secured a high degree of familiarity between the authors and the focus group participants [36]. Both positive and negative findings are presented. The authors of this study encompassed a diverse group of researchers from different disciplines. Both nurse and physician facilitators from two different simulation centres contributed to multiple interprofessional perspectives [36]. Only nine out of 20 facilitators invited chose to participate. Limited time was stated as cause for not participating. Not all facilitators participated in every focus group interview, although they did all take part in testing newly developed debriefing techniques. Still this variability in participation may have contributed to less varied viewpoints. Consolidated Criteria for Reporting Qualitative Research (COREQ) guided the reporting of this study [37].

## Conclusion

Facilitators identified and tested 22 debriefing techniques that potentially could address complexity and resilience in IPSE. Seventeen techniques were found to be useful in making students aware of the complex nature of interprofessional teamwork and collaboration in acute dynamic healthcare situations. The recommendation of two of these techniques was contingent upon time constraints and the facilitator’s workload. Five techniques were rejected. A major finding is that the adopted techniques may increase students’ awareness of their existing capacities for resilience and potentially also expand their ability to meet the need for resilience in teamwork.

Further studies are needed to explore how both students and professionals respond to resilience-focused debriefing techniques and to evaluate the effects of these techniques on interprofessional teamwork in simulation-based education.

## Data Availability

No datasets were generated or analysed during the current study.

## Abbreviations

IPSE: Interprofessional simulation-based education
DBR: Design-based research
CRM: Crisis resource management
ABCDE: Airway, breathing, circulation, disability, exposure/environment
